# Intratracheal Amylase Levels and Salivary Aspiration in Children with Tracheostomy

**DOI:** 10.31662/jmaj.2023-0213

**Published:** 2024-06-03

**Authors:** Hitomi Kubota, Hiroyuki Iijima, Noriko Morimoto, Akira Ishiguro

**Affiliations:** 1 Center for Postgraduate Education and Training, National Center for Child Health and Development, Tokyo, Japan; 2 Department of General Pediatrics and Interdisciplinary Medicine, National Center for Child Health and Development, Tokyo, Japan; 3 Division of Otorhinolaryngology, National Center for Child Health and Development, Tokyo, Japan

**Keywords:** amylase, aspiration, children, laryngotracheal separation, tracheostomy

## Introduction

Children with tracheostomy often find difficulty in swallowing owing to underlying conditions, including neuromuscular diseases or chromosomal abnormalities ^(1)^. Tracheal aspiration of saliva is a leading cause of pneumonia and results in an increased burden on caregivers. In the medical care of children with tracheostomy, management of saliva and intratracheal secretions is important ^(2)^. Aspiration of saliva is usually evaluated by fiberscopic examination, esophagography, or dye testing, but when the amount of aspirated saliva is small, detecting aspiration with these methods is challenging. Under these situations, only a few exceptionally skilled physicians can assess salivary aspiration. Therefore, developing a method to easily evaluate saliva aspiration is highly desirable.

Amylase is an easily measured digestive enzyme secreted in saliva, and higher intratracheal amylase levels would be expected in patients with frequent aspiration. Nevertheless, few studies have explored the presence of amylase in the endotracheal secretions themselves. Whether aspiration can be assessed by measuring amylase in the trachea remains unclear. In this study, we investigated intratracheal amylase levels in patients with laryngotracheal separation and evaluated the ability of intratracheal amylase measurements to assess salivary aspiration.

## Methods

We carried out a retrospective descriptive study of children (<18 years of age) with tracheostomy who visited the National Center for Child Health and Development (NCCHD) from March 2002 to August 2022. Patients with tracheal amylase measurements were included. Using a suction tube, tracheal secretions were obtained through the tracheostomy stoma. Intratracheal amylase was measured using commercially available kits (Sicafite AMY-G7, Kanto Chemical, Tokyo). We collected the following data from the electronic medical records: medical history, sex, age at measurement of intratracheal amylase, tracheal amylase level, age at tracheostomy, age at laryngotracheal separation, laryngotracheal separation technique, observation period, and frequency of aspiration pneumonia requiring antimicrobial therapy. The observation period was from the most recent tracheostomy or laryngotracheal separation. Categorical variables are expressed as numbers or percentages, and continuous variables are expressed as medians with ranges. Informed consent was obtained from patients’ guardians via an opt-out website. The Ethics Committees of the NCCHD approved this study in December 2022 (No. 2022-154).

## Results

From March 2002 to August 2022, we identified 11 children with tracheostomy who had tracheal amylase measured. Table 1 summarizes the clinical characteristics and tracheal amylase levels. The median age at the measurement of intratracheal amylase was 10 years (range 0-17 years). The median levels of intratracheal amylase ranged from 103 to 115,500 U/L. Case 6 had blood, salivary, and intratracheal amylase levels measured simultaneously, with values of 93,600, 1160, and 64 U/L, respectively. Intratracheal amylase levels were measured under normal conditions, not during the acute phase of infection. The patients were 54% female. All patients had underlying diseases, including cerebral palsy, Pfeiffer syndrome, and skeletal dysplasia. The median observation period was 4.9 years (range 1.2-11.1 years), and the median frequency of pneumonia requiring antimicrobial therapy was 0.5/year (range 0.0-3.0/year). Using tracheal flap closure, a surgical technique for preventing aspiration in pediatrics, nine patients (82%) underwent laryngotracheal separation ^(3)^. Two patients (Cases 10 and 11) had congenital laryngeal atresia.

**Table 1.** Clinical Characteristics and Laboratory Data of Cases with Tracheostomy.

Case	Age	Sex	Underlying disease	Amylase in trachea (U/L)	Frequency of pneumonia (/year)	Observation period (years)	Age at tracheostomy	Age at laryngotracheal separation
1-1	12.2-13.1	Female	Pfeiffer syndrome	115,500 (103,900-1,321,000)	3	5.6	0.2	7.8
1-2	13.8	Female	Pfeiffer syndrome	103	1.7	1.2	0.2	13.3
2	17.7	Male	Cerebral palsy	3176	0.2	5.4	2.0	12.2
3	17.9	Female	Cerebral palsy	1680	0.6	10.7	9.6	9.6
4	12.4	Female	Lissencephaly	7150	0.5	11.1	3.7	3.7
5	10.7	Male	Cerebral palsy	830	0.2	4.8	8.3	8.3
6	5-6.1	Male	Skeletal dysplasia	1320 (1160-2530)	0	4.3	1.1	2.4
7	8.4-8.5	Female	CHARGE syndrome	1575 (1010-2140)	0	3.2	4.5	7.3
8	7.8	Female	Severe neonatal asphyxia	11,100	0.5	1.8	0.3	7.3
9	14.2	Male	Congenital cytomegalovirus infection	1259	0.5	8.2	5.3	6.6
10	2.6	Male	Congenital laryngeal atresia	300 (74-336)	0.2	4.9	0	None
11	1.3	Female	VATER/VACTERL syndrome	2330	0.7	4.5	1.2	None

### Case 1

The patient was a 14-year-old bedridden girl with Pfeiffer syndrome. She was born by cesarean section at 39 weeks of gestation, and her birth weight was 3250 g. She underwent a tracheostomy at 3 months of age due to vocal cord paralysis and upper airway narrowing. At 7 years old, she underwent her first laryngotracheal separation because of repeated aspiration pneumonia. The method of laryngotracheal separation was as follows: A tracheal flap was created and sutured to the posterior wall; then, the anterior cervical muscle was placed over the flap. Despite the laryngotracheal separation, endotracheal secretions remained high, and she frequently presented with respiratory distress symptoms. Fiberscopic examination identified intratracheal granulation, managed with adjustment of the tracheal cannula and topical corticosteroids. This granulation was refractory to the treatments, and her airway symptoms did not improve. At 12 years old, she was admitted to our hospital with a fever and airway mucus hypersecretion. Laboratory data indicated an elevated white blood cell count (22,370/μL, neutrophils 90.8%) and C-reactive protein (2.22 mg/dL), with normal renal and liver functions. A chest radiogram showed consolidation at her left lower lung field. Although the blood cultures were negative, tracheal sputum culture revealed the presence of *Pseudomonas aeruginosa*, α-hemolytic *Streptococcus*, and *Corynebacterium* spp. Pneumonia was diagnosed and treated with amoxicillin-clavulanate for 5 days. With antimicrobial therapy, her fever and laboratory data improved, but her airway secretions remained high. During hospitalization, amylase levels in saliva and the trachea were 115,500 and 103,900 U/L, respectively. At 13 years old, a fiberscopic examination revealed a granulation on the posterior tracheal wall but no salivary flux. The cause of the increased endotracheal secretions was unknown. As a retest of her tracheal amylase showed a high level (1,321,000 U/L), salivary aspiration was suspected. A fiberscopic re-examination has shown salivary flux via the fistula in the posterior tracheal wall below the closure site with the granulation. After revised laryngotracheal separation using tracheal flap closure, the amount of her airway secretions and the frequency of suctioning decreased. The intratracheal granulation disappeared, and the tracheal amylase level decreased to 103 U/L.

## Discussion

This study suggests that intratracheal amylase measurement is helpful in the evaluation of aspiration. In Case 1, we found a salivary influx from the suture site of the initial laryngotracheal separation. Nevertheless, identifying the salivary flux via fiberscopic examination was challenging because a granulation had formed in exactly the same location. The high tracheal amylase level led to suspicion of aspiration of saliva, and an additional fiberscopic examination identified saliva influx, leading to revised surgery. After laryngotracheal separation, the intratracheal amylase level decreased dramatically. The preoperative intratracheal amylase level, which was close to the salivary amylase level, might have represented aspiration of saliva.

Previous studies of orally intubated adult patients reported that intratracheal amylase levels help to assess aspiration ^(4), (5)^. By contrast, intratracheal amylase values in pediatric patients with laryngotracheal separation have rarely been reported. The primary function of amylase is to cleave starch into smaller polysaccharides during digestion. The pancreas and salivary glands have much higher amylase concentrations than other tissues. These two organs are probably responsible for almost all serum amylase activity in normal individuals ^(6)^. There is a report that lung inflammation, such as due to pneumonia and lung cancer, leads to hyperamylasemia in blood, lung, and pleural effusion. However, whether amylase is secreted in the trachea is unclear ^(7)^. Until now, in a single study examining amylase in intratracheal secretions without saliva contamination, 16 elderly laryngectomized patients had a median level of 295 U/L of intratracheal amylase (range 35-1125 U/L) ^(8)^. In this study, we found amylase in the trachea of patients who underwent laryngotracheal separation. Although the function of amylase in the trachea is unclear, these data suggest that amylase is secreted into the trachea.

This study had some limitations, that is, we examined only 11 patients whose underlying diseases varied. The normal range of intratracheal amylase levels is unclear, and determining clearly whether the high intratracheal amylase levels were due to saliva aspiration or intratracheal secretion is challenging. However, our Cases 6 and 7 had amylase levels of 1010-2530 U/L, but no history of pneumonia, and the range of intratracheal amylase levels in elderly laryngectomized patients was reported to be 35-1125 U/L ^(8)^. Additionally, a previous study of human cadavers has reported that no salivary amylase was detected in the trachea ^(9)^. These results suggest intratracheal amylase levels of approximately 0-2000 U/L may be considered intratracheal secretion without aspiration. By contrast, if the amylase levels in the intratracheal secretions are very high at a level comparable to saliva, as in our Case 1, this should be considered as salivary aspiration. An important limitation is that demonstrating precisely whether or not there was aspiration in Cases 2-11 has not been possible. We could only determine aspiration in Case 1. Therefore, evidence in this study is limited. To establish a normal range for intratracheal amylase levels and to clarify its function, additional studies of intratracheal amylase are required. We suggest that intratracheal amylase measurement could help in the identification of aspiration, which is challenging if conducted via other methods, including fiberscopic examination, esophagography, and dye testing. Moreover, this method may be useful in detecting aspiration for children who are uncooperative during fiberscopic examination.

## Article Information

### Conflicts of Interest

None

### Author Contributions

Hitomi Kubota: Data collection, analysis, and writing first draft

Hiroyuki Iijima: Methodology, supervision, writing, review, and editing

Noriko Morimoto: Methodology, supervision, writing, review, and editing

Akira Ishiguro: Methodology, supervision, writing, review, and editing

### Approval by Institutional Review Board (IRB)

No. 2022-154 National Center for Child Health and Development
